# Progress and challenges of midwifery education in Indonesia

**DOI:** 10.18332/ejm/142496

**Published:** 2021-10-29

**Authors:** Qorinah Estiningtyas Sakilah Adnani

**Affiliations:** 1 Department of Public Health, Faculty of Medicine, Universitas Padjadjaran, Bandung, Indonesia

**Keywords:** COVID-19, midwifery education, midwifery practice, national framework, Indonesia

**Dear Editor,**

The COVID-19 pandemic across the globe has dramatically impacted midwifery education in Indonesia as well. The profound changes in midwifery education that have taken place include the transfer of all face-to-face classes to online platforms, which has created other challenges due to inadequate internet resources and more. The simulation and practice held in the laboratory before clinical placement has to be replaced by online learning, which is considered different from the real situation. The challenges continue with clinical placement, as midwifery students need to have hands-on experience with real women and families at maternity services. Due to the spread of COVID-19 among the community, there has been anxiety and uncertainty among midwifery students, midwifery lecturers, and midwives’ mentors during clinical placement, especially as 300 midwives have died from COVID-19 in Indonesia^1,2^.

Van Lerberghe et al.^3^ provided compelling evidence that educated, licensed and regulated midwives from high-quality midwifery education programs play a pivotal role in countries burdened with high maternal mortality rates, such as Indonesia^3^. Over the past several decades, Indonesia has employed maternal health programs by increasing the number of midwives to reduce maternal mortality rates^3^. However, Indonesia did not meet the goal of reduction by three-quarters of maternal mortality rates during the Millennium Development Goals period (2000–2015)^4^.

Indonesia, a country where every three minutes a child aged under five years dies (about 150000 per year)^5^, has a long history of midwifery practice and midwifery education^6^. In 2018, 62.7% of maternity services at childbirth were provided by midwives, followed by obstetricians-gynaecologists (28.9%), general practitioners (1.2%), and nurses (0.3%), with 93.1% of childbirths overall being assisted by skilled attendants and 6.2% being provided by traditional birth attendants^7^.

Midwives can obtain registration by completing either a three-year diploma of midwifery or a five-year professional midwifery program. The midwifery curriculum has a balance of clinical practice to theory in the ratio of 60:40 to produce a level of graduates that meets international standards and national guidelines. To date, the Indonesian government has licensed 856 private and public institutions offering midwifery programs^6-8^.

The road to the 2019 Midwifery Act started 150 years ago, with the establishment of midwifery schools in Indonesia by the Dutch colonial government. The Midwifery Act is vital in ensuring a national framework of midwifery education in Indonesia. This framework means that the professional midwifery program is now the only route to becoming an independent midwife and getting a midwifery licence to open a private midwifery practice^6^.

The provision of high-quality midwifery education in Indonesia remains a challenge for the future. Future work is required, for example, to develop a Midwifery Council as a professional regulatory board. A Midwifery Council would ensure midwives meet and maintain professional midwifery education standards, including accreditation of midwifery programs in the future and high-performance by midwives throughout their years in midwifery practice^6^. Myriads of challenges in midwifery education will remain and must be addressed to ensure new midwives’ competencies in Indonesia.

## Data Availability

Data sharing is not applicable to this article as no new data were created.
